# Epigenetic roles of PIWI proteins and piRNAs in lung cancer

**DOI:** 10.1186/s13578-019-0368-x

**Published:** 2019-12-21

**Authors:** Hadis Fathizadeh, Zatollah Asemi

**Affiliations:** 1 Department of Microbiology, Kashan University of Medical Sciences, Kashan, Islamic Republic of Iran; 2 Research Center for Biochemistry and Nutrition in Metabolic Diseases, Kashan University of Medical Sciences, Kashan, Islamic Republic of Iran

**Keywords:** PIWI protein, PiRNA, Lung cancer

## Abstract

Lung cancer is one of very important malignancies which are related to high mobility and mortality in the world. Despite several efforts for improving diagnosis and treatment strategies of lung cancer, finding and developing new and effective therapeutic and diagnostic are needed. A variety of internal and external factors could be involved in lung cancer pathogenesis. Among internal factors, epigenetic mechanisms have been emerged as very important players in the lung cancer. Non-coding RNAs is known as one of epigenetic regulators which exert their effects on a sequence of cellular and molecular mechanisms. P-element induced wimpy testis (PIWI)-interacting RNAs (piRNAs or piR) is one of small non-coding RNAs that the deregulation of these molecules is associated with initiation and progression of different cancers such as lung cancer. Several activities are related to PIWI/piRNA pathway such as suppression of transposons and mobile genetic elements. In vitro and in vivo studies demonstrated the upregulation or downregulation of PIWI proteins and piRNAs could lead to the increasing of cell proliferation, apoptosis reduction and promoting tumor growth in the lung cancer. Hence, PIWI proteins and piRNA could be introduced as new diagnostic and therapeutic biomarkers in the lung cancer therapy. Herein, we have focused on PIWI proteins and piRNA functions and their impact on the progression of lung cancer.

## Introduction

Lung cancer is one of very important malignancies which are related to several public health problems [1]. Non-small cell lung cancer (NSCLC) is known as main type of lung cancer which includes 85% of lung cancers, and small-cell lung cancer is the rare type of lung cancer which includes 15% of them [2]. Lung cancer has a poor prognosis and is often diagnosed when patient is in advanced stages of the disease, so the mortality rate of this cancer is high [3]. Treatments that are commonly used for lung cancer include surgery resection, radiotherapy and chemotherapy, are not able to significantly increase the survival rate of patients [4]. It has been showed that a wide range of internal (i.e., genetic and epigenetic factors) and external factors are involved in the lung cancer pathogenesis. Increasing knowledge about genetic, environmental and epigenetic factors can help to discover the mechanisms which are involved in the lung cancer development, including invasion and progression [5]. Identification of the pathogenesis of lung cancer epigenetically can play an important role in discovering new diagnostic biomarkers and also in developing therapeutic strategies. Epigenetic changes including DNA methylation, histone modifications, and microRNA patterns have critical roles in cancer progression [6]. Non-coding RNAs is a class of RNAs which have a big function in different physiological and pathological conditions. MicroRNAs, long non-coding RNAs, circular RNAs and piRNAs are known as various types of non-coding RNAs [7].

PiRNAs are a class of non-coding RNAs with 26 to 31 nucleotides in length. Silencing the gene expression through a mechanism of interaction with nucleotides is one of the most common activities of non- coding RNAs [8]. It is often associated with the involvement of a member of the argonaute family, which is connected to the non-coding RNAs. Argonaute proteins can be phylogenetically divided into two subclasses based on their sequence similarities. The largest subfamily includes argonautes (AGO), named based on Ago1 proteins of *Arabidopsis thaliana*, and the second subfamily is the PIWI, named based on the PIWI proteins of *Drosophila melanogaster* which are responsible for maintenance of germline stem-cell and self-renewal in *Drosophila* [9–11]. Repression of transposons leads to genomic stability, and targeting mRNAs are functions which are related to PIWI/piRNA pathway [12, 13]. For example, PIWIL4 (MIWI2) is embedded in the nucleus and directly suppresses the retrotransposons through methylation of their promoter sequences during de novo DNA methylation in male mice before birth. The loss of methylation of retrotransposon promoters has been observed in mouse testes by creating mutation in MIWI2 and MIWI [14, 15]. Also, mutations in the PIWI/piRNA pathway could increase double-stranded DNA breaks in germ cells of *Drosophila* and this increase in DNA damage results in upregulation of transposons [16, 17]. Another study indicated, when the catalytic domain of PIWIL1 (MIWI) is mutated, although it has no effect on the biogenesis of piRNA, but increases the retrotransposons in mouse testes [18]. PiRNAs interfere with the process of division stem cells, apoptosis, epigenetic regulation of telomeres and transposons, and translation control via suppression of transposable elements [19–21]. In addition, piRNAs are also found in somatic cells and act through induction of DNA methylation and histone modifications [22, 23]. Different studies have reported that upregulating piRNAs in various cancers such as ovarian cancer and pancreatic cancer [13]. It has also been shown that high expression of PIWI proteins in stomach, endometrium, gastro-intestinal tract and breast cancer cells leads to increase in tumor growth and progression than normal cells [24, 25]. Several studies have been conducted the role of piRNA and PIWI proteins in lung cancer [26–28]. Since understanding of the pattern of expression of these molecules and identification of their role in various stages of lung cancer can lead to early diagnosis and survival of the patient, we decided to review findings in this area and have a conclusion about the role of PIWI proteins and piRNA in lung cancer.

## PIWI proteins in lung cancer

PIWI proteins are highly conserved in terms of structure and activity in a wide diversity of organisms [29]. *Drosophila* has three PIWI encoding genes including PIWI, Argonaute3 (Ago3), and Aubergine (Aub) [11]. These genes in mice are PIWIl1, PIWIl2, and PIWIl4 [30]. PIWI proteins contain four members in humans: PIWIL1 (HIWI), PIWIL2 (HILI), PIWIL3 (HIWI3) and PIWIL4 (HIWI2) [29] (Table 1). Today, it is known that the PIWI subfamily bind to piRNAs and they have limited expression patterns that contain germline and mature stem cells, whereas AGO proteins bind to both miRNAs and siRNAs and extensively expressed in animal tissues [12]. Obtained information showed that the activity of PIWIs is related to mature piRNA in order to form the piRNA-induced silencing complex (piRISC), which by silencing of transposable elements helps to maintain the integrity of genome [20]. PIWI proteins are involved in various cancers by inhibiting cell growth suppressants, maintaining proliferative signals, mediating instability of genome, mutation, stimulating invasion and metastasis, and increase in cell growth [31]. Studies conducted on PIWI family revealed that it can be considered as a diagnostic, prognostic and therapeutic biomarkers for all types of cancers [32, 33]. Tumor progression and metastasis are correlated with DNA methylation rate, so that methylation rate in tumor tissue is lower than normal tissue, and the reduced DNA methylation leads to mitotic recombination and chromosome deletions and translocations, which increases chromosome rearrangements [34]. PIWI proteins and piRNA exert their influence by causing epigenetic changes in transposon elements and chromatin structures such as DNA methylation. PIWI/piRNAs pathway and epigenetic changes in tumors are likely to affect the ability of metastasis to tumorigenesis [35]. One study analyzed the expression of PIWI proteins in during lung embryogenesis and in both tumor and normal tissue resected of NSCLC patients [27]. Results showed that patterns of PIWI expression during lung organogenesis are different and specific for each PIWI gene. Also, PIWI genes that were detected in tumor and normal lung tissues exhibited different expression patterns [27]. It was found that PIWIL1 has a high expression in 7-week embryos, which follow up with a downregulation in subsequent weeks of growth [27]. PIWIL1 expression was also observed in tumor samples, but was not seen in any of the normal specimens. Patients with PIWIL1 expression had a shorter time to relapse (TTR) and overall survival (OS) than patients without PIWIL1 expression.

**Table 1:** *Drosophila* melanogaster, murine and human PIWI genes

Gene	Organism	Location	Nuclear or cytoplasmic location (reference)
Aubergine (aub)	*Drosophila melanogaster*	Chr2L	Cytoplasmic [59]
Argonaute3 (ago3)	*Drosophila melanogaster*	Chr3L	Cytoplasmic [59, 60]
Piwi	*Drosophila melanogaster*	Chr2L	Nuclear [61, 62]
Piwil1 (Miwi)	*Mus musculus*	Chr5 (5 G1.3)	Cytoplasmic [30, 63]
Piwil2 (Mili)	*Mus musculus*	Chr14 (14 D2)	Cytoplasmic [63]
Piwil4 (Miwi2)	*Mus musculus*	Chr9 (9 A2)	Nuclear [63]
Piwil1 (hiwi)	*Homo sapiens*	Chr12 (q24.33)	Nuclear/cytoplasmic [32, 64]
Piwil2 (hili)	*Homo sapiens*	Chr8 (p21.3)	Nuclear/cytoplasmic [65]
Piwil3 (hiwi3)	*Homo sapiens*	Chr22 (q11.23)	Nuclear/cytoplasmic [64]
Piwil4 (hiwi2)	*Homo sapiens*	Chr11 (q21)	Nuclear/cytoplasmic [64]

PIWIL4 was downregulated in tumor tissues and patients with lower levels of PIWIL4 had a shorter TTR and OS than others [27]. The epigenetic activities of PIWI are related to piRNAs and also, the piRNAs are able to silence the transposons by DNA methylation. In this regard, PIWIL4 could modify chromatin through methylation in the p16Ink4a locus, which it confirms positive relation between PIWIL4 levels and DNA methylation [27]. Another study, Qu et al. [36] revealed that PIWIL2 was greatly expressed in NSCLC tissues compared with normal tissues. They also reported a significant negative relation between the PIWIL2 and OS and disease-free survival [36]. MTT assay and flow cytometry indicated that increasing the PIWIL2 expression induces the proliferation and inhibits apoptosis in H460 and A549 cell lines [36]. In addition, the PIWIL2 expression may lead to the increasing of CDK2 and cyclin A expression both at protein and mRNA levels, which are related to tumorigenesis in nude mice.

CDK2 and cyclin A are two key factors in controlling the synthesis of DNA and cell cycle, and their absence lead to inducing the apoptosis and cell death [36]. Overall, it was documented that PIWIL2 participates in the progression of CDK2 and cyclin A [36]. Other studies indicated that the suppression of PIWIL1 expression via plasmids containing short hairpin RNA (shRNAs) was associated with a decrease in the cell proliferation and inducing apoptosis of lung cancer stem cells [28, 37]. Therefore, it was suggested that the *PIWIL1* gene could be considered as a molecular target for the treatment of lung cancer, and the use of the *PIWIL1* gene silencing technology is considered as a promising treatment [28, 37]. In an investigation, the PIWIL1 expression was manipulated to evaluate its role in the proliferation of NSCLC [38]. Researchers examined the PIWIL1 expression at both levels of protein and mRNA of samples collected from 57 patients with NSCLC by gain of function and loss of function strategies. The proliferation of human A549 cell line was also evaluated using colony formation assays and cell counting kit-8 [38]. Results showed that protein and mRNA expression levels of the PIWIL1 had considerably upregulation in NSCLC specimens. Also, the knockout and overexpression of the PIWIL1 were associated with the inhibition and promotion of cell proliferation, respectively [38]. Xie et al. [39] showed that the PIWIL1 overexpression facilitated proliferation, migration and invasion in lung cancer cells. Given that DNA hypomethylation of the PIWIL1 promoter is one of the factors contributing to its abnormal expression in tumors. Then, they examined the relationship between the PIWIL1 expression and mutations in lung adenocarcinoma, and eventually found that the PIWIL1 expression was remarkably higher in patients who did not have serine/threonine kinase 11 (STK11) or hepatocyte growth factor (HGF) mutations [39]. Thus, they reported that high expression of the PIWIL1 may be dependent on its promoter methylation, but not mutation dependent [39].

In a microarray study, it showed that RASSF1C, a tumor growth and migration promoter, modulated the expression of several genes that are contributed in cancer progression, cell proliferation, cell growth, cell cycle, and cell death [40]. Also, it demonstrated that the PIWIL1 had the high expression in lung cancer cell lines compared with normal cells. The overexpression of RASSF1C induces ERK1/2 phosphorylation in lung tumor cells, and prohibition of MEK–ERK1/2 pathway represses the PIWIL1 gene expression [40]. Therefore, the RASSF1C can exert its function on the PIWIL1 through MEK–ERK1/2 pathway activation [40] (Table 2). Regarding changes in PIWI expression in lung cancer, PIWI can be considered as a diagnostic biomarker and a therapeutic target for the management of lung cancer in order to further investigation.

**Table 2:** Experimental studies that investigated the role of PIWI proteins in lung cancer

PIWI	Up/Down	Tissue/cell line	Technique	Final result	Ref
PIWIL1	Up	H23 and A549 cell lines	Real-time PCR/immunohistochemistry/ELISA assay	Decrease in survival time of patients and shorten time to relapse	[27]
PIWIL4	Down	H23 and A549 cell lines	Real-time PCR/immunohistochemistry/ELISA assay	Increase of transposon activities and genomic instability/decrease in survival time of patients and shorten time to relapse	[27]
PIWIL2	Up	A549 and H460 cell lines/nude mice	Real-time PCR/western blot/immunofluorescence staining/immunohistochemistry/flow cytometry	Proliferation induction/tumor growth promoting/apoptosis inhibition	[36]
HIWI	Up	SSC^lo^ Alde^br^ cells/SPC-A1 cell line/mice	Hiwi shRNA plasmids treatment/immunohistochemistry/enzyme immunoassay/flow cytometry	Increasing proliferation/apoptosis reduction	[28, 37]
HIWI	Up	A549 cell lines	Real-time PCR/western blot/gain of function and loss of function strategies/colony formation assay/cell counting assay	Cell proliferation increasing	[38]
PIWIL1	Up	A549 and H1299 cell lines	Real-time PCR/gain of function and loss of function strategies/western blot/bioinformatics analysis	Enhancing proliferation, migration and invasion	[39]
PIWIL1	Up	A549/NCIH1299/CRL- 9482	Microarray analysis/real-time PCR/western blot	Increase in cell growth and proliferation	[40]

## PiRNAs in lung cancer

Although PIWI proteins are very important for carcinogenesis, piRNAs have a considerable role in this process. In reproductive tissues, piRNAs are abundantly expressed. In addition, piRNAs expression has been observed in the brain, and human plasma-derived exosomes also have about 1.31% piRNAs [41]. Unlike microRNAs that are involved in post-transcriptional regulation, most piRNAs are more associated with epigenetic regulation for controlling various biological processes such as angiogenesis, invasive, tumor growth and metastases [42, 43]. The epigenetic changes in including histones hypoacetylation, DNA hypomethylation, and gene-specific hypermethylation can lead to oncogene function and silencing of tumor suppressors [43, 44]. So far, several piRNAs have been found that are associated with cancer progression such as piR-932, piR-823, piR-651 [26, 45, 46]. PiR-651, one of the members of the piRNA family, involves in carcinogenesis via interacting with HIWI [47]. Cheng et al. [24] demonstrated that piR-651 expression upregulates in hepatic carcinoma, lung, colon, gastric, breast, mesothelioma and stomach cancer cell line by piRNA microarray and real-time RT-PCR. They also suggested that the HIWI has a significant role in cancer development and may be as a potential target in cancer therapy. In this study, cell cycle analysis showed that piR-651 inhibitor can stop cancer cells in G2/M phase. In fact, piRNA pathway has a role in cell division and self-renewal equilibrium, which a disruption in this balance causes an important effect in cancer progression [24].

In another study, piR-651 expression levels were assayed by RT-qPCR, in situ hybridization and northern blot test [26]. Results showed piR-651 expression upregulated in NSCLC that this abnormal expression was related to tumor progression in patients with NSCLC. Also, the overexpression of piR-651 causes a remarkable enhance in viability and metastasis rate in cancer cell line [26]. After piR-651 upregulation, percentage of stopping cells in G0/G1 phase was less than control. Moreover, cyclin D1 and CDK4 expression levels were correlated with rate of piR-651 expression both in vitro and in vivo [26]. Using nude mice and injection of piR-651 containing plasmids, in order to create a xenograft model, it was revealed that there was a correlation between the increasing of the piR-651 expression and tumor development, which is mediated by cyclin D1 and CDK4. Authors concluded that piR-651 could be considered as diagnostic marker and therapeutic target for lung cancer therapy [26]. Furthermore, a study conducted on 95-D lung cancer cells showed that the piR-651 expression in cancer cells was higher than normal cells [48]. This investigation using of different assays such as trans well and wound-healing tests, MTT and flow cytometry demonstrated that piR-651 exerts its regulating roles on cancer via influencing cell apoptosis, cell proliferation, migration and metastasis [48]. Zhang et al. [49] reported that applying of the piR651 inhibitor and its transfection into HCC827 and NSCLC A549 cell line can lead to the inhibition of cell proliferation and remarkably increasing of the rate of apoptosis, decreasing of the number of migrating cells than the control group as well as altering apoptosis-associated proteins expression levels. In general, these studies concluded that the piR651 can be used as an important diagnostic biomarker and an effective therapeutic target in lung cancer [48, 49].

In some cases, the piRNAs expression was decreased in different cancers, which lead to increasing of cancer cell proliferation and tumor progression [50]. For example, the piR-55490 expression was downregulated in lung cancer and various studies had shown that the recovery of the piR-55490 can decline the proliferation rates of lung cancer cells, while suppression of piR-55490 leads to enhance the proliferation rates [51]. The piR-55490 suppresses the function of Akt/mTOR pathway in lung cancer cells. Indeed, piR-55490 binds to 3′UTR of mTOR mRNA and reduces its decay by a mechanism like miRNAs. The existence of the piRNA can lead to repression of tumor cell phenotypes through a targeting of oncogene mRNA [51]. In an investigation on *DLK1*-*DIO3* locus at a chromosome interesting, results were obtained about piRNA expression [52]. *DLK1*-*DIO3* locus is related to promoting respiratory disorders such as lung cancer. It is able to encode many genes, including protein-coding genes, long non-coding RNAs and short non-coding RNAs [52]. Finding demonstrated that the piRNAs expression encoded at *DLK1*-*DIO3* increases the prognostic ability of sncRNAs related to this locus in order to predicting patients with lung cancer outcomes [52]. Furthermore, Reeves et al. [53] reported the identification of the piRNAs in lung adenocarcinoma cells over-expressing the RASSF1C. They found that piR-52200 and piR-34871 were up-regulated, while piR-46545 and piR-35127 were down-regulated in half of tested tumor tissues. Microarray screen and real time PCR confirmed that the expression of this piRNAs regulates by the RASSF1C [53]. The knockdown of the piR-52200 and piR-34871 and the overexpression of piR-46545 and piR-35127 remarkably decreased the proliferation of H1299 and A549 cell lines. So, they concluded that these piRNAs can be involved in regulating of transformation and tumorigenesis of lung cell and, the RASSF1C may modulate piRNAs target genes expression by the attenuation of AMPK pathway [53].

## PiR-Ls in lung cancer

Recently, PIWI-interacting RNA likes (piR-Ls) have been reported as genetic elements that can regulate their target phospho-proteins (p-protein) in lung cells. However, their mechanism of action is still unclear [54]. In fact, the greatest difference of piR-Ls from other ncRNAs is that piR-Ls can directly bind to its target (p-proteins), but do not follow base-pairing rules [54]. One of differences between piR-Ls and piRNAs is their limited size, for example, piR-L-163 has 30 nucleotides and piR-L-138 with 29 nucleotides, while piRNAs have a range of 26 to 32 nucleotides in mammalian cells [55, 56]. In addition, piR-Ls have new sequences and were detected in adult tissues [56].

It has recently been reported that piR-Ls can play a significant role in different physiological and pathological conditions of lung [55, 56]. In a study, patterns of piRNA/piRNA-L expression were examined in human lung bronchial epithelial (HBE) and NSCLC cells [55]. Findings indicated that piR-L-163 can be involved in the cell growth, proliferation, invasion and migration through directly binding to phosphorylated ERM proteins and regulating of ERM function [55]. The ERM is containing ezrin, radixin and moesin proteins which belong to a group of proteins placed at the cell membrane. They have a key role in the regulating signal transduction pathways [57]. PiR-L-138 is one of another piRNA-like small RNAs that has critical role in tumorigenesis of lung cancer [56]. PiR-L-138 was upregulated at chemoresistance to cisplatin (CDDP)-based chemotherapy in vitro and in vivo [56]. Targeting piR-L-138 led to increasing the apoptosis in both CDDP-treated cell line and xenograft mice. Mouse double minute 2 homolog (MDM2) and its isoforms implicate in p53-independent apoptosis and in chemoresistance [58]. PiR-L-138/p60-MDM2 interaction causes inhibition of CDDP-activated apoptosis in p53 mutants [56]. So, discovering piR-Ls function and enhancing knowledge about its capabilities can provide a potential strategy to the overcome resistance to chemotherapy in patients with lung cancer [56] (Table 3).

**Table 3:** Experimental studies that investigated the role of piRNAs in lung cancer

PiRNAs/PiR-Ls	Up/Down	Related pathway	Tissue/cell line	Final result	Ref
PiR-651	Up	Cyclin D1 and CDK4 pathway	HepG2/HeLa/Bcap-37/MSTO- 211H/NCI-H446/MGC-803/SGC-7901/95-D/NSCLC A549/HCC827/A549 cells	Increase in cancer progression and cell viability/promoting metastasis, invasion and migration/apoptosis reduction	[24, 26, 48, 49]
PiR-55490	Down	Akt/mTOR pathway	A549/H460/H1299/MRC-5	Increase in cell proliferation and tumor progression	[51]
PiR-34871 and piR-52200	Up	AMPK pathway	A549/H1299	Cell proliferation enhancing	[53]
PiR-46545 and piR-35127	Down	AMPK pathway	A549/H1299	Cell proliferation enhancing	[53]
PiR-L-163	Down	No investigated	H157/H226/H596, SK-MES-1/H522/H1437/H1792/H1944/HBE2/HBE3/HBE4	Increasing of cell growth, invasion, and migration	[55]
PiR-L-138	Up	piR-L-138/p60-MDM2 phosphorylation	CDDP-based chemotherapy treated LSCC cell lines/PDX LSCC models with a CDDP based regimen	Apoptosis inhibition	[56]

## Conclusions

**Fig. 1:**
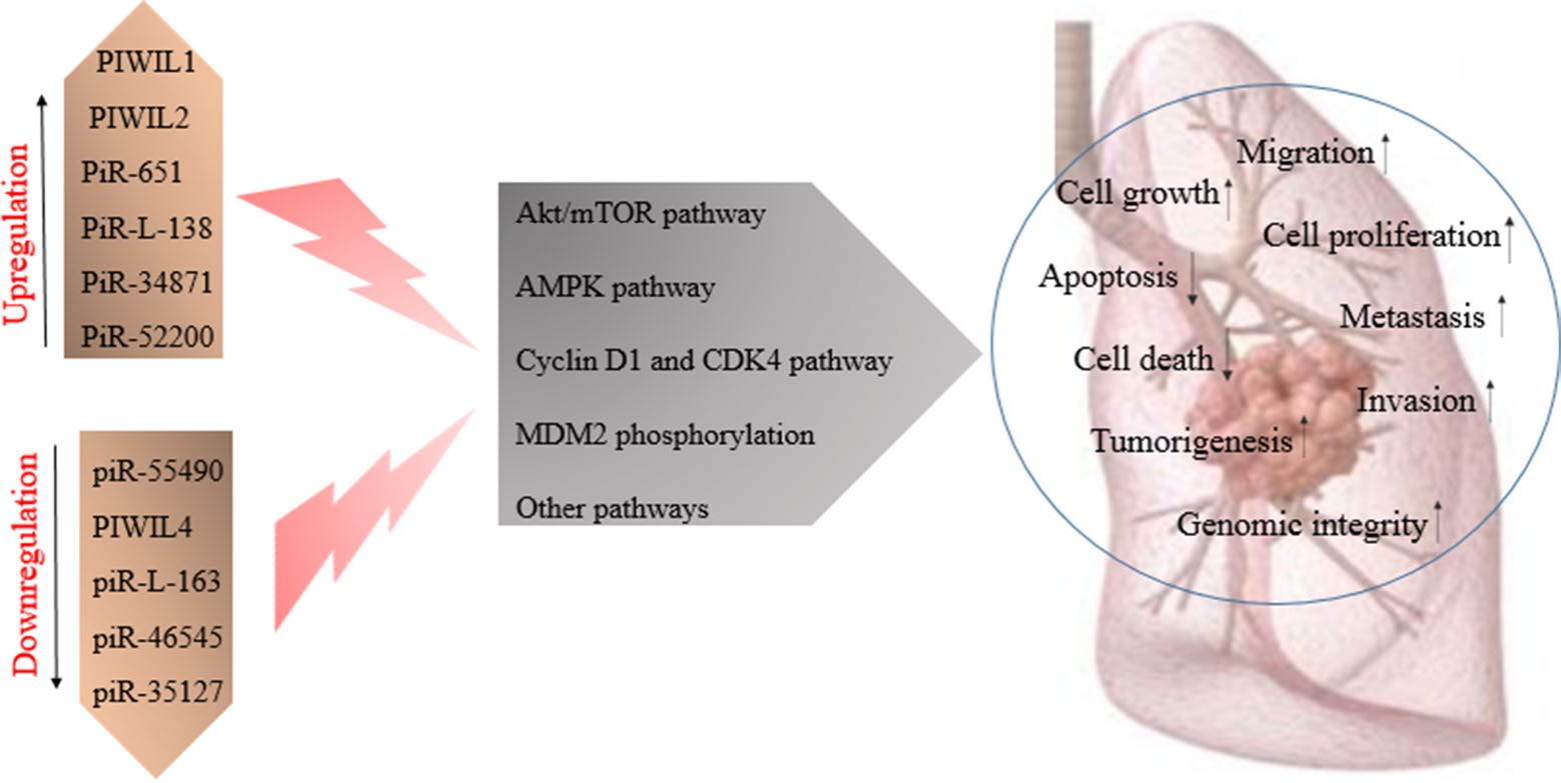
Factors related to PIWI proteins and piRNAs in lung cancer

Lung cancer is one of the leading causes of death in the world, and the incidence and deaths from this disease is increasing. Unfortunately, lung cancer is commonly diagnosed in the late stages of the disease, and common therapies, including surgery, do not contribute to the complete improvement of patients, therefore the attention of researchers has been drawn to finding new diagnostic and therapeutic approaches, including the use of molecular biomarkers. In recent years, studies of non-coding small RNA containing siRNA, miRNA and piRNA are increased to find appropriate therapeutic and diagnostic approaches. An increasing number of reports have demonstrated the abnormal expression of PIWI and piRNA in different cancers such as breast, colon, gastric, ovarian, bladder and lung cancer, and it confirmed that PIWI proteins can be involved in tumorigenesis and progression of cancer. Disturbance in PIWI-piRNAs pathway regulation and its effect on cancer-related biological processes, including proliferation, apoptosis, invasion, migration and metastasis, suggests that PIWI proteins and piRNAs can be used as diagnostic biomarkers and therapeutic targets for the treatment of lung cancer (Fig. 1). So, manipulating PIWI proteins and changing the rate of PIWI and piRNAs gene expression can lead to cancer control and patient recovery.

## Data Availability

Not applicable.

## Abbreviation

PIWI: P-element induced wimpy test
